# Are Psychotic Experiences Related to Poorer Reflective Reasoning?

**DOI:** 10.3389/fpsyg.2018.00122

**Published:** 2018-02-12

**Authors:** Martin J. Mækelæ, Steffen Moritz, Gerit Pfuhl

**Affiliations:** ^1^Department of Psychology, University of Tromsø, Tromsø, Norway; ^2^Department of Psychiatry and Psychotherapy, University Medical Center Hamburg-Eppendorf, Hamburg, Germany

**Keywords:** delusions, schizophrenia, decision making, rationality, psychosis continuum, autism, asperger syndrome

## Abstract

**Background:** Cognitive biases play an important role in the formation and maintenance of delusions. These biases are indicators of a weak reflective mind, or reduced engaging in reflective and deliberate reasoning. In three experiments, we tested whether a bias to accept non-sense statements as profound, treat metaphorical statements as literal, and suppress intuitive responses is related to psychotic-like experiences.

**Methods:** We tested deliberate reasoning and psychotic-like experiences in the general population and in patients with a former psychotic episode. Deliberate reasoning was assessed with the bullshit receptivity scale, the ontological confabulation scale and the cognitive reflection test (CRT). We also measured algorithmic performance with the Berlin numeracy test and the wordsum test. Psychotic-like experiences were measured with the Community Assessment of Psychic Experience (CAPE-42) scale.

**Results:** Psychotic-like experiences were positively correlated with a larger receptivity toward bullshit, more ontological confabulations, and also a lower score on the CRT but not with algorithmic task performance. In the patient group higher psychotic-like experiences significantly correlated with higher bullshit receptivity.

**Conclusion:** Reduced deliberate reasoning may contribute to the formation of delusions, and be a general thinking bias largely independent of a person's general intelligence. Acceptance of bullshit may be facilitated the more positive symptoms a patient has, contributing to the maintenance of the delusions.

## Introduction

Most of us have had moments saying silent or aloud “how the … can you believe in this bullshit?” Is this person joking with me or is the person meaning it seriously? If it is the latter, should you worry? Given that psychotic symptoms are not just experienced by patients with a psychiatric disorder but are also present in a larger portion of the general population would receptivity to bullshit be a cognitive marker for bizarre thoughts and non-conventional logic?

We here investigated whether receptivity to bullshit, a tendency to confabulations, and a general reduction in cognitive reflection are positively associated with more psychotic-like experiences.

A plethora of research has shown that mental disorders are related to failures in rationality (Zimbardo, 1999; Adams et al., 2016), i.e., knowledge of when and how to apply deliberate reasoning (Stanovich, 2009). A primary example is psychosis and psychosis-like experiences. Psychotic-like experiences (PLE) are subclinical delusional ideas and perceptual disturbances, with psychosis being the pathological extreme (Poulton et al., 2000; van Os et al., 2009). PLE are qualitatively similar to Symptoms experienced by patients with psychosis but quantitatively less severe with lower frequency, intensity and persistence. These experiences occur in 5–8% of the general population (van Os et al., 2009), but among adolescents the incident rate is higher (Scott et al., 2008; van Os et al., 2009; Kelleher and Cannon, 2011). PLE are associated with the development of schizophrenia spectrum disorders as well as a number of other mental health problems such as anxiety, depression, substance abuse, self-harm and suicide risk (van Os et al., 2002; Hides et al., 2009; Saha et al., 2011a,b). PLE can be seen as an alteration in how one perceives and thinks about reality, and similar to delusions, which are a cardinal feature of psychosis, they present bizarre or abnormal thoughts and non-conventional logic. PLE can therefore, as delusions, be seen as examples of epistemic irrationality, although less severe.

### Models explaining the formation of PLE and delusions

One model explaining positive symptoms in psychosis is the Dual Stream Modulation failure model by Speechley and Ngan (2008). This model looks at decision-making as a product of the dynamic interaction between a fast, intuitive and automatic form of processing (Stream 1) and a slower, conscious and deliberative process (Stream 2). In scenarios where there is more than one option available the detection of conflict tends to shift decision-making toward Stream 2. Highly emotionally valenced stimuli tend to shift decision-making toward Stream 1. This model proposes that in schizophrenia there is a failure in conflict detection and hence a reduced shift of decision-making toward Stream 2 and an increased (emotional) shift toward Stream 1. This aberrant interaction of the two streams facilitates erroneous intuitive explanations to coexist with contrary logical explanations of the same event contributing to delusions.

Another model explaining the positive symptoms of psychosis by Moritz et al. (2017) outlines a two-stage process. Stage 1 or the belief formation stage is how false ideas enter and dominate consciousness. Patients tend to assign meaning and momentum to weakly supported evidence. Patients tend to show a greater acceptance of absurd hypotheses in psychological paradigms, i.e., they show a lowered decision threshold referred to as liberal acceptance bias (Moritz and Woodward, 2004; Moritz et al., 2006, 2009, 2016; Veckenstedt et al., 2011). At stage 1 the delusion is more like a working hypothesis, and delusions in this stage are usually not acted upon. Stage 2, or belief maintenance stage, consists of several factors promoting conviction (McLean et al., 2016).

Indeed, PLE and delusions may arise through a faulty interplay between the more rational, conscious, slow, serial, and cognitively demanding system 2 (which promotes deliberate reasoning), and the intuitive, fast, unconscious system 1 (responsible for intuitive thinking; Evans, 1984; Sloman, 1996; Kahneman, 2003; Speechley and Ngan, 2008; Speechley et al., 2013).

Further, Stanovich (2009) propose a model separating the execution of deliberate reasoning and the knowledge of when to apply this. In his tripartite model the reflective mind is responsible for knowing when to apply deliberate reasoning, while the algorithmic mind (i.e., intellectual capacity) is responsible for the execution of reasoning.

Accordingly, a weak reflective mind can lead to impaired conflict detection, and monitoring of ideas and beliefs. This may lead individuals to accept questionable information, draw premature conclusions, or fail to challenge and recalibrate their beliefs (Balzan et al., 2016). Our proposition is therefore that PLE and epistemic irrationality are due to a weakened reflective mind, while algorithmic capacity may be intact and normal in persons with PLE.

A different type of model, the diametric brain hypothesis (Crespi and Badcock, 2008), sees psychosis and autism as diametrically opposite phenotypes especially in the social domain. This model suggests hypermentalizing in PLE but hypomentalizing in autism. Of the deliberate reasoning tasks we are using (see below) confabulating and acceptance of bullshit might be linked to hypermentalizing and hence autistic traits may protect against too much confabulation and bullshit. However, a recent study showed that individuals high on both autistics and psychotic traits had normal mentalizing abilities, while individuals high on either measure alone had decreased performance (Abu-Akel et al., 2015). We therefore included a brief measure of autistic traits in Experiment 3.

### Assessing the reflective mind—measuring deliberate reasoning

Recently, Pennycook et al. (2015) developed a task measuring uncritical acceptance, the bullshit receptivity task. The colloquial term *Bullshit* refers to statements that are created to impress but are not related to truth (Frankfurt, 2005). One may want to impress others with technical or complex terms despite insufficient knowledge (Atir et al., 2015). Bullshit is a statement purporting to be deep and impressive but is merely buzzwords placed together with correct syntactical structure (Pennycook et al., 2015). The tendency to perceive such nonsense statements as profound has been labeled “bullshit receptivity” (BR). BR can be explained by two mechanisms. The first is a bias to accept things at face value. The second is a lack of ability to detect bullshit, or an error in the discovery of meaninglessness. BR is shown to correlate positively with supernatural beliefs, conspiracy theories and religiosity, which is epistemic questionable faith (Pennycook et al., 2015). A further indicator of a weak reflective mind is the tendency to confabulate. Ontological confabulation (OC) (Lindeman et al., 2015) is any category mistake involving property differences between “animate” and “inanimate” or “mental” and “physical.” One example of OC is confusing biological and psychological phenomena such as “An evil thought is contaminated.” Ontological confabulations are seen as a failure to reflect on and inhibit intuitive confusions of ontological phenomena (Svedholm and Lindeman, 2013). It has been shown that paranormal believers mentalize matter, physicalize and biologize mental phenomena more than skeptics do (Lindeman and Aarnio, 2007). One can also look at OC as a bias toward believing the literal truth of statements. In this way, it could be related to BR, and indeed has been shown to correlate positively with it Pennycook et al. (2015). A well-established measurement of the reflective mind is the Cognitive Reflection Test (CRT) (Frederick, 2005). It is designed to test an individual's ability to override an intuitive, but mistaken response with a more analytical correct response. The CRT has been shown to correlate negatively with receptivity for bullshit and ontological confusion (Pennycook et al., 2015).

Together, these three tasks serve as a measure of the reflective mind, and can give insight into the mechanisms contributing to PLE. It should be noted that BR and OC are lexical tasks, and depend on verbal reflective abilities, while the CRT relies more on reflective numeracy (Thomson and Oppenheimer, 2016). We also included two short tests measuring verbal and numerical abilities, the wordsum test (Malhotra et al., 2007) and the Berlin numeracy test (Cokely et al., 2012). A fourth task that may measure a weak reflective mind is a probabilistic inference task, i.e., the beads task (Huq et al., 1988). Patients with psychosis request less information before deciding, referred to as “jumping to conclusion” bias (JtC bias).

In line with Speechley and Ngan's (2008) Dual Processing Modulation Failure account, we expect a high score of PLE to correlate negatively with CRT performance. Further, by the liberal acceptance account (Moritz and Woodward, 2004) and the diametrical brain hypothesis (Crespi and Badcock, 2008) we expect that people with higher levels of PLE will be more receptive to bullshit and display more ontological confabulations. We expected no relationship between PLE and algorithmic task performance. Experiment 1 was an explorative study, whereas Experiment 2 set out to confirm the findings in patients that have a diagnosis of psychosis. Experiment 3 replicated Experiment 1 and added a measure of autistic traits.

## Experiment 1: exploring reflective mind measures and psychotic experiences

### Method participants and procedure

Eighty-five people (34 men) from UiT The Arctic University of Norway and Sunnhordland Folkehøgskole participated. The participants were undergraduate psychology students, undergraduate law students, and undergraduate music students. The mean age was 21 years (*SD* = 3.5). Participation was anonymous, fully voluntary and with informed consent. Participants were informed that the investigation focused on problem solving in everyday life. The survey was implemented in Qualtrics (Qualtrics, Provo, UT) and completed by participants in a computer lab at the campus. It took ~30 min to complete. Participants received no compensation.

#### Tasks and material

Following demographic assessment participants completed three tasks to test deliberate reasoning (i.e., their reflective mind), two tasks to gauge their algorithmic thinking and two trials of the fish task, which is a variant of the beads task (see below).

#### Deliberate reasoning tasks (reflective mind)

The Cognitive Reflection Test (CRT) is designed to test an individual's ability to override an intuitive but incorrect response in order to correctly solve a mathematical problem (Toplak et al., 2011, 2014; Thomson and Oppenheimer, 2016). An example of an item is “A bat and a ball cost $1.10 in total. The bat costs $1 more than the ball. How much does the ball cost? We used items 2–6 from Toplak et al. (2011), and scored answers as either correct or incorrect. Correct answers were coded as 1 and incorrect answers as 0, resulting in a score from 0 to 5 for the 5 tasks.

Bullshit receptivity (BR) was measured with the bullshit receptivity scale containing 10 non-meaningful items such as “We are in the midst of a self-aware blossoming of being that will align us with the nexus itself” and a motivational scale with 10 meaningful sentences “All endings are also beginnings. We just don't know it at the time.” Pennycook et al. (2015) used a Five Point Likert scale, but we used a 4-point scale from 1 = “not deep at all” to 4 = “very deep meaning” to avoid people using the middle score as a “don't know”-option (Dolnicar and Grün, 2014).

Ontological Confabulations (OC) were measured with the 14 items from the Core Knowledge Confusions scale. Participants were asked to rate statements such as “When summer is warm, flowers want to bloom” on a four-point scale from 1 = completely metaphorical to 4 = completely literal. This was supplemented with three metaphorical items like “Friends are the salt of life,” and three literal items “Running water is fluid” (Lindeman et al., 2015). The confabulation score is the sum of participants' responses to the 14 Core Knowledge Confusion items. The higher the score the more they treated those statements as literal.

#### Algorithmic thinking tasks

Participants completed a short numeracy and a short verbal intelligence test. This controlled also for collinearity to ensure that the measures of deliberate reasoning were separate from a person's IQ.

The Berlin Numeracy Test (Cokely et al., 2012) uses four mathematical tasks that become progressively more difficult. Participants only proceeded from one question to the next item if they answered the previous question correctly. Correct answers were awarded 1 point and incorrect answers 0 points, for a possible total score ranging from 0 to 4.

Wordsum is a short test of verbal intelligence (Malhotra et al., 2007). The task presents 10 words, each with 5 alternatives of which one is a synonym. One point was awarded for each correctly identified synonym. It was possible for participants to answer “I don't know.” The final score ranges between 0 and 10 points. We applied an exclusion criterion of <3 points (chance level is 2 out of 10 correct). No participant was excluded due to a too low score.

The fish task (Speechley et al., 2010) was included to test whether there is a relationship between the deliberate reasoning tasks and a jumping to conclusion bias. The task consists of two lakes being presented on a screen, and the participants are asked to decide from which lake a black (b) or white (w) fish comes from, respectively. We used two rounds with 10 draws. In round one the ratio of black to white fish was 80:20 in lake A and 20:80 in lake B and the sequence was bbbwbbbbwb. In round two the ratio of black to white fish was 80:20 in lake A and 50:50 in lake B and the sequence was wbwbbwbwwb. Participants saw one fish at a time and could indicate from which lake the fish comes from, or choose to see another fish before deciding. They also adjusted two sliders to indicate the probability that the fish came from lake A and lake B, respectively^1^. How many fish they required to see before making a decision is the “draws to decision” score (DtD). Deciding after one or two fish is considered jumping to conclusion. Participants who did not decide on a lake after 10 draws were scored as making a decision on the 11th draw.

Finally, we measured the rate of PLE with the 20 items from the positive scale of the CAPE-42 (Stefanis et al., 2002), abbreviated as CAPE-P. A sample item is “Do you ever feel as if electrical devices such as computers can influence the way you think?” A second scale measures the distress associated with these experiences, together the two scales produce a sum total score of PLE. The distress scale was only used in Experiments 1 and 2.

The order of the tasks was first CRT, then BR task, OC task, wordsum task, numeracy task, then the two rounds of the fish task and lastly the CAPE-P.

We expected to find positive correlations between the CAPE-P and the BR, CAPE-P and the OC as well as we expected to find a negative correlation between draws to decision and CAPE-P, the CRT and the CAPE-P (predictions i–iv). These four predictions were corrected for multiple comparisons with the False discovery rate (Benjamini and Hochberg, 1995).

Further, auxiliary^2^ predictions were a negative relationship between CRT and BR score as well as CRT and OC; a positive correlation between BR and OC (Pennycook et al., 2015) and a positive correlation between the Berlin numeracy test score and the CRT (Cokely et al., 2012; prediction iv–viii). Data analysis was performed with JASP (JASP Team, 2017). We also performed a regression analysis with CAPE-P score as dependent variable and the scores from the six tasks (CRT, BR, OC, wordsum, Berlin numeracy test, fish task's draws to decision) as predictors. The decision threshold from the fish task was not included.

### Results

Fourteen participants received a survey version without the fish task, and due to a technical problem their data on the CRT got lost.

The correlation table below (Table 1) provides all 21 correlations. Prediction (i–ii): There was a significant positive correlation between CAPE-P and BR score and CAPE-P and the OC score, (iii) but not between CAPE-P and the draws to decision score, i.e., the draws to decisions in the two rounds of the fish task was not related to the number of psychotic-like experiences. Prediction (iv–vi): There was no significant correlation between CAPE-P and CRT or CRT and BR score, but there was a significant negative correlation between CRT and OC score. Prediction (vii) was met, i.e., the OC score had a significant positive correlation with the BR score. Prediction (viii) was met, i.e., the Berlin numeracy score had a significant positive correlation with the CRT score. In addition, we noted that the wordsum score was significantly negatively correlated to the BR score. The wordsum score and the numeracy score were not correlated with the CAPE-P. We noted also an unpredicted result, a higher score in the wordsum task had a significant positive correlation with more draws to decision and a reduced JtC bias, respectively.

**Table 1:** Pearson correlation among the six tasks and the CAPE-P score.

		**Confabulation score**	**CRT score**	**Berlin Numeracy test**	**Wordsum score**	**DtD /JtC**	**CAPE_P + distress**
Bullshit receptivity score	Pearson's r	**0.332**	−0.151	0.099	−**0.266**	−0.042	**0.299**
	*p*-value	**0.002**	0.208	0.365	**0.014**	0.726	0.006^*^
Confabulation score	Pearson's r	—	−**0.301**	−0.139	−0.162	−0.169	**0.305**
	*p*-value	—	**0.011**	0.205	0.138	0.158	0.005^*^
CRT_score, *n* = 71	Pearson's r		—	**0.287**	0.028	−0.005	−0.123
	*p*-value		—	**0.015**	0.814	0.965	0.305
Numeracy test	Pearson's r			—	0.056	0.097	−0.029
	*p*-value			—	0.608	0.420	0.793
Wordsum score	Pearson's r				—	**0.249**	−0.118
	*p*-value				—	**0.036**	0.282
Draws to Decision (JtC bias), *n* = 71	Pearson's r					—	−0.151
	*p*-value					—	0.207^t^

Note that for CRT and for DtD N = 71 only, FDR corrected p-values are 0.012 for ^*^, and P = 0.276 for ^t^ in bold: correlations with p_uncorrected_ < 0.05.

A stepwise linear regression for the CAPE-P score yielded two significant predictors: bullshit receptivity score [*t*_(84)_ = 2.038, *p* = 0.045, CI:.016–1.275] and confabulation score [*t*_(84)_ = 2.124, *p* = 0.037, CI:.710–21.619]. This regression explained 14% of the variance in the CAPE-P scores (*p* = 0.002).

### Discussion

We found a positive correlation between a person's receptivity to bullshit, ontological confabulation and the number of PLE. We did not find a relationship between the CRT and PLE among healthy young persons, nor was there a negative relationship between the CRT and the bullshit receptivity score, but there was a negative relationship between the CRT and the confabulation score. Since the bullshit receptivity score and the confabulation score were positively correlated, this suggests that these two tasks measure one aspect of the reflective mind and the CRT another aspect, i.e., lexical and numerical deliberate reasoning, respectively. Good verbal intelligence is necessary but not sufficient for the BR, OC and fish task. The CRT score might depend on numerical ability (Thomson and Oppenheimer, 2016) and not only on the reflective mind. The two algorithmic thinking tasks, wordsum and numeracy, are unrelated to each other. We found no relationship between the draws to decision in the fish task and PLE. This is not surprising since we used only two rounds, and it came after a series of other tasks. The task is prone to misunderstanding of the instructions (Balzan et al., 2012) and higher verbal intelligence was here associated with a lower JtC bias. In addition, recent meta-analyses have found only weak support for the JtC bias and PLE in non-deluded persons (Ross et al., 2015; Dudley et al., 2016), presumably due to motivational issues/noise in the healthy controls (Moutoussis et al., 2011).

## Experiment 2: deliberate reasoning in patients with schizophrenia

In Experiment 1 we found that the number of PLE and associated stress were positively related to a person's acceptance of nonsense statements and interpretation of metaphorical statements as literal. The difference between people with PLE and schizophrenia is foremost quantitative (more PLE) and then becomes qualitatively different. As such, a weakened reflective mind may be a cognitive marker for symptom severity. Hence, we wanted to see whether symptom severity among patients with a diagnosis of psychosis is related to bullshit receptivity and ontological confabulation. We expected to find a positive correlation between symptom severity and acceptance of bullshit, more ontological confabulations, and a lower score in the CRT.

### Methods

#### Participants and procedure

Thirty seven people^3^, whereof 19 were patients took part. The patients were recruited from a database of participants derived from former studies carried out at the University Medical Center Hamburg-Eppendorf (Germany). They were provided information about the experiment and the link to the survey via email, i.e., we did not know who of the contacted patients participated. Inclusion criteria were a lifetime diagnosis of psychosis or schizoaffective disorder, age of 18–65 years, informed consent, and (because all assessments were conducted online) availability of a web-enabled smartphone or a personal computer with internet access throughout assessment (Lüdtke et al., 2017). Contacted patients were currently not in an acute stage but we do not know whether they were currently unwell^4^. Eighteen people of the same age as the patient group were recruited through snowballing. Exclusion criterion was any mental disorder diagnosis, but we did not pre-select for participants with a low PLE. After completing the tasks, participants could watch a video of the northern lights with relaxing music. No monetary incentives were given.

#### Tasks and material

The tasks were the same as in Experiment 1 except that the fish task was not included. The survey was implemented using Qualtrics and it took ~20 min to complete.

### Results

The groups were well matched by age, sex, and education. As can be seen in Table 2, they differed in their CAPE-P score but not in the deliberate reasoning or algorithmic thinking tasks.

**Table 2:** Demographics and task performance between controls and patients.

**Demographics/tasks**	**Controls (*N* = 18, 11 female)**	**Patients (*N* = 19, 13 female)**	***P***	**Cohen's d**
Age	42.9 ± 15.5	44.5 ± 11.2	0.714	0.122
Education	3.0 ± 0.84	2.53 ± 0.84	0.096	0.563
CAPE-P + distress^*^	35.83 ± 6.32	51.32 ± 16.49	< 0.001	1.24
Bullshit Receptivity	19.33 ± 5.22	17.68 ± 6.55	0.405	0.277
Ontological Confabulation	32.94 ± 7.53	31.37 ± 8.19	0.547	0.2
Cognitive Reflection Test	2.83 ± 1.72	2.32 ± 1.25	0.301	0.345
Wordsum score	7.83 ± 1.43	8.63 ± 1.74	0.137	0.501
Berlin numeracy score	1.22 ± 1.35	1.05 ± 1.35	0.705	0.125

* *Welch test, data is M ± SD*.

Within the patient group we found a significant strong positive correlation between CAPE-P and BR score: *r* = 0.512, *p* = 0.025, but not for the OC score: *r* = 0.199, *p* = 0.415. The CRT score was not significantly related to the CAPE-P score, *r* = 0.003, *p* = 0.99. As found in Experiment 1, there was a significant high correlation between the CRT and the numeracy test score among the 19 patients, *r* = 0.647, *p* = 0.003. BR score and OC score were not related, *r* = −0.052, *p* = 0.834 (see Table 3).

**Table 3:** Pearson correlation between the five tasks and the CAPE-P in 19 patients.

		**Confabulation score**	**CRT_score**	**Berlin Numeracy test**	**Wordsum score**	**CAPE_P + distress**
Bullshit receptivity score	Pearson's r	−0.052	−0.293	−0.142	−0.245	**0.512**
	*p*-value	0.834	0.224	0.561	0.312	0.025^*^
Confabulation score	Pearson's r	—	−0.224	−0.087	−0.372	0.199
	*p*-value	—	0.357	0.723	0.117	0.415
CRT_score	Pearson's r		—	**0.647**	0.414	0.003
	*p*-value		—	**0.003**	0.078	0.990
Numeracy test	Pearson's r			—	0.268	−0.095
	*p*-value			—	0.266	0.698
Wordsum score	Pearson's r				—	0.041
	*p*-value				—	0.867

*In bold: correlations with p_uncorrected_ < 0.05*,

* *FDR corrected p = 0.05*.

### Discussion

Patients with at least one previous psychotic episode reported more PLE and associated distress than age-matched controls. Within the patient group severity of symptoms correlated positively with BR score but did not correlate with the OC score, CRT score, or reduced algorithmic thinking as measured with the wordsum task and the numeracy task. Further, patients did not differ from controls on any of these tasks which might be due to our small sample size. A clear limitation is that we had no information on the duration, medication or comorbidity in the patient sample. These findings complement Experiment 1, as PLE positively correlates with BR in the patient sample but BR is not a sensitive enough measurement to distinguish patients from controls. However, BR and not OC correlated with severity of the symptoms.

## Experiment 3: deliberate reasoning along the autistic-psychosis continuum

In Experiments 1 and 2 we used only the positive subscale of the CAPE-42. Notably, the OC is a mentalizing task, and the BR might be so too, i.e., what is an agent telling me? Additional autistic traits might counterbalance such hypermentalizing in persons with many PLE. Indeed, a recent study found that healthy participants scoring high on the CAPE-P and also scoring high on the autism spectrum quotient (AQ) performed as good as participants scoring low on both scales in a perspective taking task (Abu-Akel et al., 2015). Hence, we reasoned that the number of autistic traits is negatively correlated with bullshit receptivity and ontological confabulations. Since the CRT is not a mentalizing task, we did not expect a positive correlation between autistic traits and CRT. To gauge depressive tendencies we used the entire CAPE-42, i.e., including the negative symptom subscale^5^.

### Methods

#### Participants

Eighty-one participants from UiT and NTNU took part, many were psychology undergraduates. Five participants had to be excluded due to answering either all of the three catch questions (see below) positively or had a wordsum score below 3. Thirteen participants did not do the confabulation task, wordsum task, Berlin numeracy task, and the CRT. The analysis for CAPE, AQ, and BR is based on *N* = 76 (54 women), whereas the analysis for the confabulation task and CRT is based on *N* = 63 (46 women). The mean age (*N* = 76) was 23.8 years (*SD* = 2.87, range 19–35).

#### Tasks and material

In Experiment 3 we introduced two new measures: the 10-item Autism spectrum quotient (AQ-10, Booth et al., 2013) and the 25-item Systematizing quotient (SQ) (Wakabayashi et al., 2006) to see if these traits could serve as a protective factor on deliberate reasoning tasks in healthy controls.

The experiment was done in two parts. Participants first completed the BR task, then the 10-item version of the AQ (Booth et al., 2013), the 25-item version of the SQ (Wakabayashi et al., 2006), and the CAPE-42 with three catch items (Moritz et al., 2013) interspersed but without the distress scale. These items were presented in a custom-written python script. Then, participants opened the browser and completed the CRT, OC task, the wordsum task, and the numeracy task in Qualtrics. All testing occurred in a computer lab at the campus and took ~35 min. Participants received no compensation.

We predicted a positive correlation between the BR score and the CAPE-P, and between the OC score and CAPE-P, but a negative correlation between AQ and the BR score and the OC score, respectively. These four predictions were corrected for multiple comparisons with the FDR. We had no prediction for the two other subscales of the CAPE-42: the depressive (CAPE-D) and negative (CAPE-N) subscale. We further expected that the CRT score is positively correlated with the Berlin numeracy score, and that the wordsum score is negatively correlated with the BR score. We predicted a positive correlation between the SQ and the numeracy score (Baron-Cohen et al., 2007).

### Results

As predicted (Table 4) we found a positive correlation between the CAPE-P and the BR score, *r* = 0.23, and the CAPE-P and the OC score, *r* = 0.234 but neither reached statistical significance, FDR corrected *p's* = 0.09. The AQ was negatively correlated with the BR score, *r* = −0.254, FDR corrected *p* = 0.09; but the AQ was not related to the confabulation score, *r* = −0.083, *p* = 0.519. Of the auxiliary predictions we found that the BR score and the OC score were significantly positively correlated, *r* = 0.339, *p* = 0.007, as was the CRT and the numeracy score, *r* = 0.46, *p* < 0.001. Contrary to prediction, the wordsum score was not correlated with the BR score, *r* = −0.067, *p* = 0.599. The SQ had a positive but non-significant correlation with the numeracy score, *r* = 0.231, *p* = 0.068 and a significant positive correlation with the CAPE-D, *r* = 0.307, *p* = 0.007. After dichotomizing participants (median split) into low and high AQ, and low and high CAPE-P, respectively, there were only six participants that scored above average on both AQ and CAPE-P; this did not allow us to do an analysis as Abu-Akel et al. did.

**Table 4:** Pearson correlation between the five tasks and the five questionnaire scales.

		**Confabulation**	**CRT**	**Berlin Numeracy test**	**Wordsum score**	**CAPE-42 positive**	**CAPE-42 depressive**	**CAPE-42 negative**	**AQ-10**	**SQ-25**
Bullshit score (*N* = 76)	*r*	**0.339**	−0.075	−0.230	−0.067	**0.230**	0.113	−0.135	−**0.254**	0.041
	*p*-value	**0.007**	0.557	0.070	0.599	0.046^*^	0.333	0.246	0.027^*^	0.725
Confabulation (*N* = 63)	*r*	—	−0.215	−**0.343**	−0.127	0.234	0.106	−0.129	−0.083	−0.118
	*p*-value	—	0.091	**0.006**	0.320	0.065^*^	0.408	0.315	0.519	0.358
CRT (*N* = 63)	*r*		—	**0.466**	0.058	−0.230	−0.057	0.099	−0.044	0.130
	*p*-value		—	**<0.001**	0.649	0.070	0.658	0.442	0.731	0.311
Numeracy test (*N* = 63)	*r*			—	−0.027	0.024	0.081	**0.328**	0.091	0.231
	*p*-value			—	0.831	0.852	0.527	**0.009**	0.480	0.068
Wordsum_score (*N* = 63)	*r*				—	−0.088	−0.038	0.072	−0.198	0.191
	*p*-value				—	0.493	0.766	0.576	0.121	0.133
CAPE-42 positive (*N* = 76)	*r*					—	**0.387**	**0.387**	0.062	−0.022
	*p*-value					—	**<0.001**	**<0.001**	0.594	0.850
CAPE-42 depressive (*N* = 76)	*r*						—	**0.650**	0.117	**0.307**
	*p*-value						—	**<0.001**	0.316	**0.007**
CAPE-42 negative (*N* = 76)	*r*							—	**0.252**	0.086
	*p*-value							—	**0.028**	0.458
Autism quotient (*N* = 76)	*r*								—	0.059
	*p*-value								—	0.615

*In bold: correlations with p_uncorrected_ < 0.05*,

* *FDR corrected P = 0.09. AQ-10 … autism quotient 10-item version, SQ-25 … systemizing quotient 25-item version*.

A multiple linear regression was performed to predict CAPE-P based on the five tasks. The model had an *R*^2^ of 0.166, *F*_(5, 62)_ = 2.262, *p* = 0.06. The strongest predictor was the CRT score, *t* = −2.18, *p* = 0.033. A similar model for AQ yielded an *R*^2^ of 0.143, *F*_(5, 62)_ = 1.896, *p* = 0.109 with the BR score as the strongest predictor, *t* = 0.2.329, *p* = 0.023. Since there is no causal relationship, we also looked at which of the five scales from the questionnaires would predict receptivity to bullshit and the ontological confabulation score, respectively. A multiple linear regression for bullshit receptivity based on the CAPE-P, CAPE-D, CAPE-N, AQ, and SQ had an *R*^2^ of 0.196, *F*_(2, 75)_ = 3.421, *p* = 0.008. Both the CAPE-P (*t* = 2.359, *p* = 0.021) and the CAPE-N (*t* = −2.405, *p* = 0.019) were significant coefficients. The AQ was a near-significant coefficient, *t* = −1.903, *p* = 0.061 whereas the SQ was non-significant, *p* = 0.919. For the confabulation score, the model had an *R*^2^ of 0.169, *F*_(5, 62)_ = 2.313, *p* = 0.055, and only the CAPE-N (*t* = −2.477, *p* = 0.016) was a significant predictor.

### Discussion

Those that reported many psychotic-like experiences classified more nonsense statements as profound and metaphorical statements as literal. This was not driven by verbal intelligence as we found no relationship between the wordsum score and the BR or OC score. Notably, participants with a low score on the AQ were more susceptible to bullshit than those who had a higher AQ score but this was not the case for the SQ. We used only the 10-item version of the AQ. Only very few participants had a score of five or more on the AQ. Similarly, only few participants had extreme PLE scores. This may explain why we could not find that having a high AQ and high CAPE-P resulted in a “normal” performance (Abu-Akel et al., 2015). Still, AQ but not SQ correlated with BR. Neither of those two scales correlated with the OC score supporting Pennycook et al. (2015) finding that bullshit receptivity is separate from ontological confabulations. Notably, in the regression analysis CAPE-P, CAPE-N, and AQ predicted BR but the OC score was not predicted by CAPE-P or the AQ. Also, a high score on systemizing did not prevent receptivity to bullshit or ontological confabulation. Finally, these results indicate that there is not a simple diametrical relationship between autism and psychosis either (Russell-Smith et al., 2013).

## General discussion

Our hypothesis was that psychotic-like experiences are related to the individual's weakened ability to know when to use deliberate reasoning. We found a positive correlation between bullshit receptivity and psychotic-like experiences, and a positive correlation between ontological confabulation and psychotic-like experiences. However, the correlations were not very high but were consistent in their magnitude and direction (positive) across the three experiments. Notably, a standard test for deliberate reasoning, the CRT, was not consistently found to be associated with PLE, it was negatively correlated only in Experiment 3. The CRT was positively associated with the Berlin numeracy task (see also Toplak et al., 2011). As predicted, performance in the algorithmic tasks showed no significant relationship with the number of psychotic-like experiences. This is an important finding because this indicates that it is not the general ability to think that is aberrant, but rather a metacognitive problem of knowing when to use deliberate reasoning especially in lexical tasks.

Comparing patients to healthy controls yielded no group differences in any of the three deliberate reasoning measures. However, within the small patient group we did find a large correlation between bullshit receptivity and psychotic-like experiences. Future studies should therefore investigate if bullshit receptivity varies with symptom severity. Notably, it is particularly these nonsense statements that were related to PLE. Hypermentalizing, common in psychosis, cannot solely explain it. The ontological confabulation task is also a task involving mentalizing but did not correlated with PLE in the patient sample nor was the OC score predicted by the CAPE-P or AQ score (Experiment 3). The CRT was not consistently linked to PLE either, thus bullshit receptivity might evoke a mentalizing part that is unique and susceptible to PLE. This may also explain the negative relationship between autistic traits and bullshit receptivity in Experiment 3. Since we did not find this for the ontological confabulation task, nor any relationship with systematizing, future studies should investigate if scoring high on AQ serves as a protective factor for bullshit receptivity for individuals also high on CAPE-P.

Regarding our deliberate reasoning measurements, it should be noted that there are two mechanisms that need to be separated. One is the previously mentioned liberal acceptance bias and the other is the ability to detect and override an intuitive response (knowing when deliberate reasoning is necessary). Bullshit as an example can be separated into a bias toward saying all items have a deep meaning (liberal acceptance), or you can look at the ability to separate between control items and bullshit items (conflict detection and override). Results from a signal detection theory approach (Supplementary Material) does indicate that the CAPE-P correlates both with a bias to assign statements' profoundness and one's ability to discriminate between bullshit and control items (named motivational items in Pennycook et al., 2015). Also, ontological confabulation is related to relying on intuition and is in large part a failure to inhibit and override intuitive thinking. Svedholm and Lindeman (2013) showed that increasing cognitive load (making people rely more on intuition) increased ontological confabulation. From our three experiments it seems that persons scoring high on psychotic-like experiences have a more liberal acceptance of statements, which partly supports the dual stream modulation failure theory (Speechley and Ngan, 2008).

Thus it seems that a liberal acceptance and the failure to know when to engage in deliberate reasoning contribute to psychotic-like experience. A failure to engage in deliberate reasoning can lead to both more ontological confabulations and a larger acceptance of sentences without any deeper meaning, commonly known as bullshit. Subsequent thought processes or beliefs can build upon these faulty assumptions, without the awareness of the individual, also the individual is less likely to later correct these mistakes. In this way, faulty beliefs and assumptions will be accepted, and further beliefs can build upon false assumptions and mistakes can accumulate. Both bullshit receptivity and ontological confabulations have previously shown positive correlations with epistemically questionable beliefs such as beliefs in the paranormal, supernatural, religion and conspiracy theories (Lindeman et al., 2015; Pennycook et al., 2015), while the Cognitive reflection test and other heuristics and bias tasks (Toplak et al., 2011) have shown negative correlations with these beliefs (Pennycook et al., 2015). Furthermore, religious believers have shown similar levels of delusional ideation as patients with psychosis, and they also showed a jumping to conclusion bias (Lim et al., 2012). It might be that bullshit receptivity and ontological confabulation share a common mechanism. This mechanism might also be involved in the emergence and maintenance of religious beliefs and psychosis, respectively. Attributional mechanisms (Bentall et al., 1994), Theory of Mind deficits (Frith, 1992), trauma (Saha et al., 2011a,b,c) and other less well studied factors may differentiate between religious believers and persons with psychosis.

## Limitations and further research

To our knowledge our study is the first that examines the link between deliberate reasoning measures and psychotic-like experiences in healthy controls and patients. It warrants replication and also a longitudinal investigation to differentiate whether a weakened reflective mind contributes to e.g., the formation of delusions or its maintenance. Experiment 2 had low power due to small sample size, which might be why we did not find any group differences on our deliberate reasoning measures. Also, we have limited knowledge of the mental history of both our patients and control group. Future research should also include other groups like people with a diagnosis of autism, OCD, or depression to investigate the specificity of the correlations found here. Training the reflective mind, as does metacognitive therapy, should reduce the receptivity to bullshit, ontological confabulations and strengthen performance in the cognitive reflection test.

## Conclusion

We found that psychotic-like experiences were positively correlated with bullshit receptivity and ontological confabulations, but not with performance in the wordsum task or in the Berlin numeracy task. This indicates that psychotic-like experiences are related to a weakened reflective mind and not to a weakened algorithmic mind. Thus, a likely contributor to psychosis is poorer reflective reasoning.

## Ethics statement

Study 1 and 3 were exempt from requiring an ethical committee, since all data collection was completely anonymous (at University campus) and complied with the Norwegian Data Security guidelines i.e., the project is not subject to notification. Study 2 was conducted exclusively online, that is, study information, informed consent, and the survey were implemented online via Qualtrics. The patient sample was recruited from a database of participants derived from former studies carried out at the University Medical Center Hamburg-Eppendorf (Germany) who were contacted via e-mail. This has been approved by the ethical committee of the German Psychological Society (DGPs). At all time, participation was voluntary, could be stopped at any time, and there was at no point any personally identifiable information collected or stored.

## Author contributions

MM and GP: Designed the study; GP: Analyzed the data; MM, GP, and SM: Wrote the article; MM, GP, and SM: Recruited participants.

### Conflict of interest statement

The authors declare that the research was conducted in the absence of any commercial or financial relationships that could be construed as a potential conflict of interest.

## Footnotes

^1^Some participants mixed up the side of the sliders, or did only adjust one slider. And the decision threshold was not correlated with any other measurement, for clarity we do not include it in the Result section.

^2^These are exempt from FDR correction, reducing the Type II error. We thank one of the reviewers for this advice.

^3^Fifty-four participants started the survey, however there were 17 dropouts due to not completing the survey.

^4^We think it is unlikely that currently unwell patients or patients in remission would have completed the tasks and responded to the email, respectively.

^5^Participants with high scores on the CAPE-P and or AQ may also have depressive symptoms.
