# Supplementary material for: Are Psychotic Experiences Related to Poorer Reflective Reasoning?
Source: Front Psychol. 2018 Feb 12;9:122. doi: 10.3389/fpsyg.2018.00122 (PMC5816266; doi:10.3389/fpsyg.2018.00122)
Supplement: Supplementary file 1 [file Table1.docx]

# Supplementary material

Contents

[Supplementary material 1](#_Toc505092034)

[Experiment 1 1](#_Toc505092035)

[Experiment 2, only patient sample 2](#_Toc505092036)

[Experiment 3 2](#_Toc505092037)

[Pooled data over all three experiments 3](#_Toc505092038)

[Data files 4](#_Toc505092039)

We also performed a signal detection theory analysis to calculate a bias and a performance score, i.e. the ability to discriminate between bullshit and motivational items (Macmillan and Creelman, 2004). Below we report the results for the CAPE-P and the two parameters from the Signal Detection Theory: discrimination score d’ and bias c to accept bullshit. Note that this analysis is not possible for the ontological items as there are too few literal items in the original scale (Lindeman et al., 2015).

## Experiment 1

**Linear Regression**

| **Model Summary** | | | | | | | | | | | |  |  |  |  |  |
| --- | --- | --- | --- | --- | --- | --- | --- | --- | --- | --- | --- | --- | --- | --- | --- | --- |
| **Model** | | **R** | | **R²** | | **Adjusted R²** | | **RMSE** | | | |  |  |  |  |  |
| 1 |  | 0.329 |  | 0.108 |  | 0.082 |  | 16.123 | | |  |  |  |  |  |  |
| **ANOVA** | | | | | | | | | | | | | | | | |
| **Model** | |  | | | **Sum of Squares** | | | | **df** | | **Mean Square** | | **F** | | **p** | |
| 1 |  | Regression | |  | 2147 | | |  | 2 |  | 1073.6 |  | 4.130 |  | **0.020** |  |
|  |  | Residual | |  | 17677 | | |  | 68 |  | 260.0 |  |  |  |  |  |
|  |  | Total | |  | 19825 | | |  | 70 |  |  |  |  |  |  |  |

| **Coefficients** | | | | | | | | | | | | | | | | | |
| --- | --- | --- | --- | --- | --- | --- | --- | --- | --- | --- | --- | --- | --- | --- | --- | --- | --- |
| **Model** | |  | | **Unstandardized** | | **Standard Error** | | **Standardized** | | **t** | | **p** | | **2.5%** | | **97.5%** | |
| 1 |  | intercept |  | 49.939 |  | 3.158 |  |  |  | 15.814 |  | < .001 |  | 43.638 |  | 56.241 |  |
|  |  | Bullshit receptivity d' |  | -5.718 |  | 2.629 |  | -0.310 |  | -2.175 |  | **0.033** |  | -10.964 |  | -0.472 |  |
|  |  | Bullshit receptivity c |  | -18.596 |  | 6.634 |  | -0.399 |  | -2.803 |  | **0.007** |  | -31.833 |  | -5.359 |  |

##

## Experiment 2, only patient sample

**Linear Regression**

| **Model Summary** | | | | | | | | | | | |  |  |  |  |  |
| --- | --- | --- | --- | --- | --- | --- | --- | --- | --- | --- | --- | --- | --- | --- | --- | --- |
| **Model** | | **R** | | **R²** | | **Adjusted R²** | | **RMSE** | | | |  |  |  |  |  |
| 1 |  | 0.503 |  | 0.253 |  | 0.160 |  | 15.113 | | |  |  |  |  |  |  |
| **ANOVA** | | | | | | | | | | | | | | | | |
| **Model** | |  | | | **Sum of Squares** | | | | **df** | | **Mean Square** | | **F** | | **p** | |
| 1 |  | Regression | |  | 1239 | | |  | 2 |  | 619.7 |  | 2.713 |  | 0.097 |  |
|  |  | Residual | |  | 3655 | | |  | 16 |  | 228.4 |  |  |  |  |  |
|  |  | Total | |  | 4894 | | |  | 18 |  |  |  |  |  |  |  |

| **Coefficients** | | | | | | | | | | | | | | | | | |
| --- | --- | --- | --- | --- | --- | --- | --- | --- | --- | --- | --- | --- | --- | --- | --- | --- | --- |
| **Model** | |  | | **Unstandardized** | | **Standard Error** | | **Standardized** | | **t** | | **p** | | **2.5%** | | **97.5%** | |
| 1 |  | intercept |  | 57.540 |  | 5.763 |  |  |  | 9.985 |  | < .001 |  | 45.323 |  | 69.756 |  |
|  |  | Bullshit receptivity d' |  | -7.566 |  | 5.064 |  | -0.330 |  | -1.494 |  | 0.155 |  | -18.301 |  | 3.169 |  |
|  |  | Bullshit receptivity c |  | -22.457 |  | 10.901 |  | -0.455 |  | -2.060 |  | 0.056 |  | -45.567 |  | 0.653 |  |

## Experiment 3

**Linear Regression**

| **Model Summary** | | | | | | | | | | | |  |  |  |  |  |
| --- | --- | --- | --- | --- | --- | --- | --- | --- | --- | --- | --- | --- | --- | --- | --- | --- |
| **Model** | | **R** | | **R²** | | **Adjusted R²** | | **RMSE** | | | |  |  |  |  |  |
| 1 |  | 0.265 |  | 0.070 |  | 0.045 |  | 0.307 | | |  |  |  |  |  |  |
| **ANOVA** | | | | | | | | | | | | | | | | |
| **Model** | |  | | | **Sum of Squares** | | | | **df** | | **Mean Square** | | **F** | | **p** | |
| 1 |  | Regression | |  | 0.518 | | |  | 2 |  | 0.259 |  | 2.751 |  | 0.071 |  |
|  |  | Residual | |  | 6.871 | | |  | 73 |  | 0.094 |  |  |  |  |  |
|  |  | Total | |  | 7.388 | | |  | 75 |  |  |  |  |  |  |  |

| **Coefficients** | | | | | | | | | | | | | | | | | |
| --- | --- | --- | --- | --- | --- | --- | --- | --- | --- | --- | --- | --- | --- | --- | --- | --- | --- |
| **Model** | |  | | **Unstandardized** | | **Standard Error** | | **Standardized** | | **t** | | **p** | | **2.5%** | | **97.5%** | |
| 1 |  | intercept |  | 1.493 |  | 0.067 |  |  |  | 22.352 |  | < .001 |  | 1.359 |  | 1.626 |  |
|  |  | Bullshit receptivity d' |  | -0.078 |  | 0.045 |  | -0.213 |  | -1.729 |  | 0.088 |  | -0.168 |  | 0.012 |  |
|  |  | Bullshit receptivity c |  | 0.050 |  | 0.065 |  | 0.095 |  | 0.770 |  | 0.444 |  | -0.079 |  | 0.179 |  |

## Pooled data over all three experiments

**Linear Regression**

| **Model Summary** | | | | | | | | | | |  |  |  |  |  |  |
| --- | --- | --- | --- | --- | --- | --- | --- | --- | --- | --- | --- | --- | --- | --- | --- | --- |
| **Model** | | **R** | | **R²** | | **Adjusted R²** | | **RMSE** | | |  |  |  |  |  |  |
| 1 |  | 0.204 |  | 0.042 |  | 0.032 |  | 0.306 | |  |  |  |  |  |  |  |
| **ANOVA** | | | | | | | | | | | | | | | | |
| **Model** | |  | | | **Sum of Squares** | | | | **df** | | **Mean Square** | | **F** | | **p** | |
| 1 |  | Regression | |  | 0.796 | | |  | 2 |  | 0.398 |  | 4.248 |  | **0.016** |  |
|  |  | Residual | |  | 18.270 | | |  | 195 |  | 0.094 |  |  |  |  |  |
|  |  | Total | |  | 19.067 | | |  | 197 |  |  |  |  |  |  |  |

| **Coefficients** | | | | | | | | | | | | | | | | | |
| --- | --- | --- | --- | --- | --- | --- | --- | --- | --- | --- | --- | --- | --- | --- | --- | --- | --- |
| **Model** | |  | | **Unstandardized** | | **Standard Error** | | **Standardized** | | **t** | | **p** | | **2.5%** | | **97.5%** | |
| 1 |  | intercept |  | 1.559 |  | 0.037 |  |  |  | 42.601 |  | < .001 |  | 1.487 |  | 1.632 |  |
|  |  | Bullshit receptivity d' |  | -0.082 |  | 0.029 |  | -0.218 |  | -2.854 |  | **0.005** |  | -0.139 |  | -0.025 |  |
|  |  | Bullshit receptivity c |  | -0.079 |  | 0.047 |  | -0.129 |  | -1.689 |  | 0.093 |  | -0.170 |  | 0.013 |  |

Overall, both the ability to discriminate between nonsense statements and motivational items (d’), as well as a general decision threshold to accept statements as meaningful (bias c) are related to CAPE-P. In the first two experiments, the bias was a stronger coefficient, whereas in the third experiment we found no significant relationship between psychotic-like experiences and the two bullshit receptivity parameters. Thus, a refinement of the task, as well as further replication is needed before one can state whether it is the bias to accept bullshit (liberal acceptance account) or a general weakness in discriminating between meaningful and meaningless statements that is reduced in persons with psychotic-like experiences.

## Data files

Jasp files can be found here: https://osf.io/w4be7/
